# Use of Point-of-care Ultrasound for the Seizing Infant: An Adjunct for Detection of Abusive Head Trauma

**DOI:** 10.5811/cpcem.2020.6.48207

**Published:** 2020-07-14

**Authors:** Jonathan Rowland, Dean Fouchia, Mark Favot

**Affiliations:** Wayne State University School of Medicine, Department of Emergency Medicine, Detroit, Michigan

**Keywords:** Ultrasound, abusive head trauma, subdural hematoma

## Abstract

**Case Presentation:**

An eight-week-old infant presented to the emergency department in cardiac arrest. Return of spontaneous circulation was obtained and the patient subsequently began seizing. Point-of-care ultrasound of the anterior fontanelle revealed an extra-axial fluid collection consistent with subdural hematoma (SDH).

**Discussion:**

Abusive head trauma is still frequently missed on initial presentation. In addition to validated screening clinical prediction rules, point-of-care cranial ultrasound can be used as a noninvasive adjunct for detection of SDH related to abusive head trauma in infants with an open fontanelle.

## CASE PRESENTATION

**Image 1:**
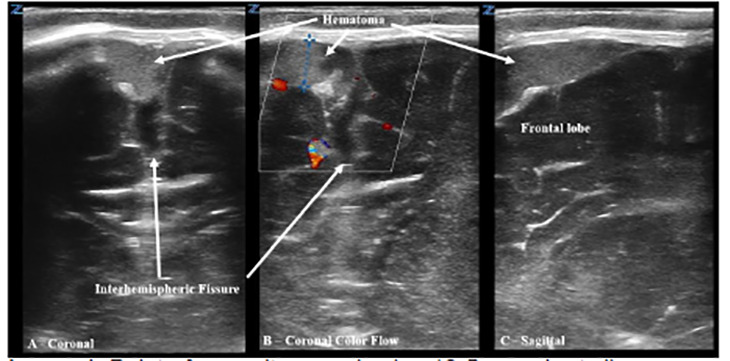
Point-of-care ultrasound using 10-5 megahertz linear probe at the anterior fontanelle demonstrating an 8-millimeter subdural hematoma in both coronal (A) and sagittal (C) orientation; and color Doppler demonstrating lack of flow (B).

An eight-week-old male with a history of laryngomalacia and prematurity born at 36 weeks presented to the emergency department (ED) in cardiac arrest after being found unresponsive at home. Cardiopulmonary resuscitation was initiated on arrival to the ED, intraosseous access was established, and the patient was intubated with subsequent return of spontaneous circulation. Ten minutes later, he had a generalized tonic-clonic seizure, and the anterior fontanelle was noted to be tense. Point-of-care ultrasound (POCUS) of the anterior fontanelle revealed an echogenic extra-axial fluid collection suspected to represent subdural hematoma (SDH) (Image 1).

**Image 2:**
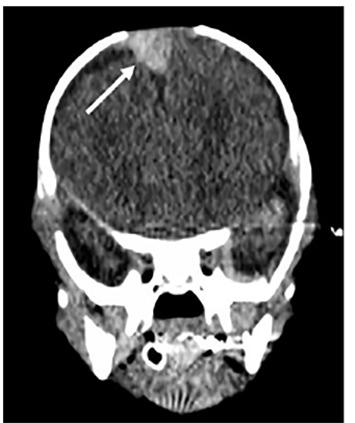
Non-contrast computed tomography demonstrating a 1.2 x 1.3 centimeter subdural hematoma (arrow).

The patient was successfully stabilized, then transferred to a tertiary center where a computed tomography (CT) of the brain confirmed the presence of an SDH, intraparenchymal hemorrhages of the hypothalamus and brainstem, and an anterior neck hematoma concerning for non-accidental trauma (Image 2).

## DISCUSSION

### Diagnosis: Acute Subdural Hematoma in Setting of Non-accidental Trauma

Abusive head trauma (AHT) is the leading cause of fatal head injuries in children under two years, with SDH being the most frequently identified lesion (up to 90%) located most commonly in the parafalcine space along the superior sagittal sinus.^1^ The rate of missed AHT remains largely unchanged for the last 20 years at roughly 30%.^2^ Best practice recommendation is to *avoid* applying PECARN head CT rule^3^ to any suspected victims of AHT, and to instead use the validated “TEN-4 FACESp”^4^ clinical prediction tool to maximize sensitivity in detection of sentinel injuries predictive of abuse.^2^ Emergency physicians have been demonstrated as capable of identifying intracerebral hemorrhage using POCUS.^5^,^6^ POCUS should be considered when evaluating infants with suspected AHT or new-onset seizures and is easily performed with brief examination of the parafalcine space through the anterior fontanelle window using a high-frequency linear probe.

**CPC-EM Capsule**

What do we already know about this clinical entity?

Subdural hematoma is the most commonly identified lesion in fatal cases of abusive head trauma (AHT). The rate of missed AHT remains largely unchanged (~30%) for the last twenty years.

What is the major impact of the image(s)?

Point-of-care ultrasound (POCUS) can be utilized in infants with open fontanelles and should be considered in evaluation of suspected AHT and/or undifferentiated new-onset seizures.

How might this improve emergency medicine practice?

POCUS can be utilized as a noninvasive adjunct, in addition to validated clinical prediction tools, to improve our detection of AHT.

## Supplementary Information

Video 1 Coronal sweep of the anterior fontanelle with a high-frequency linear 10-5 MHz probe utilizing color doppler to evaluate flow around the subdural hematoma within the interhemispheric fissure/parafalcine space.

Video 2 Coronal sweep of the anterior fontanelle at the interhemispheric fissure/parafalcine space with a high-frequency linear 10-5 MHz probe with B-mode imaging, demonstrating a subdural hematoma to the right of midline with associated widening of the interhemispheric fissure.

Video 3 Sagittal sweep of the anterior fontanelle at the frontal lobe with a high-frequency linear 10-5 MHz probe with B-mode imaging, demonstrating an echogenic subdural hematoma.
